# Supplementary figures and images for: Sitagliptin Is More Effective Than Gliclazide in Preventing Pro-Fibrotic and Pro-Inflammatory Changes in a Rodent Model of Diet-Induced Non-Alcoholic Fatty Liver Disease
Source: Molecules. 2022 Jan 22;27(3):727. doi: 10.3390/molecules27030727 (PMC8838637; doi:10.3390/molecules27030727)

S1

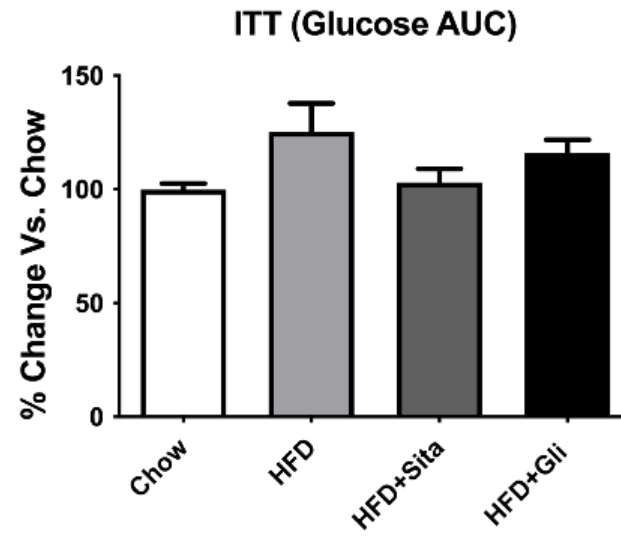

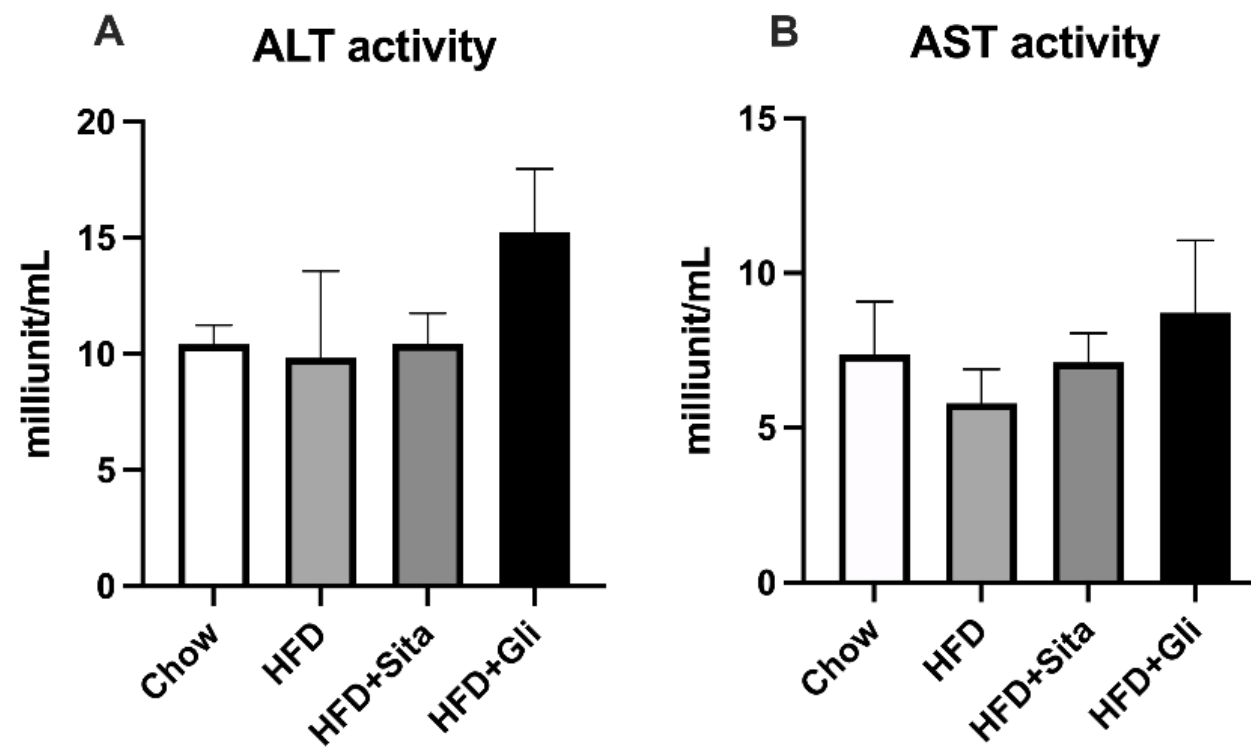

Supplement: Supplementary file 1 [file molecules-27-00727-s001.zip › molecules-1547959-supplementary.pdf]
